# Effect of interleukin 6 –174G>C gene polymorphism on opioid requirements after total hip replacement

**DOI:** 10.1007/s00540-016-2167-4

**Published:** 2016-04-05

**Authors:** Monika Białecka, Alina Jurewicz, Anna Machoy-Mokrzyńska, Mateusz Kurzawski, Katarzyna Leźnicka, Violetta Dziedziejko, Krzysztof Safranow, Marek Droździk, Andrzej Bohatyrewicz

**Affiliations:** Department of Pharmacokinetics and Therapeutic Drug Monitoring, Pomeranian Medical University, Powstańców Wlkp 72, Szczecin, 70-111 Poland; Department of Orthopaedics, Traumatology and Orthopaedic Oncology, Pomeranian Medical University, Unii Lubelskiej 1, Szczecin, 71-252 Poland; Department of Experimental and Clinical Pharmacology, Pomeranian Medical University, Powstańców Wlkp 72, Szczecin, 70-111 Poland; Department of Human Functional Anatomy and Biometry, Institute of Physical Culture and Health Promotion, Szczecin University, al. Piastów 40b, Szczecin, 71-065 Poland; Department of Biochemistry and Medical Chemistry, Pomeranian Medical University, Powstańców Wlkp 72, Szczecin, 70-111 Poland

**Keywords:** Interleukin 6, Opioids, Osteoarthritis, Polymorphism, Pain

## Abstract

**Objective:**

In recent years, increasing attention has been paid to the contribution of genetic factors to variability in patient pain threshold and the efficacy of pain management. One of the genes implicated in pain pathology and treatment response is interleukin 6 (*IL6*). The aim of the present study was to evaluate the association between *IL6* (rs1800795: –174G>C) and opioid requirements in patients after total hip replacement (THR).

**Methods:**

A total of 196 patients eligible for the study (126 women, 70 men) were subjected to THR. The THR procedure was performed using spinal anaesthesia after implementing routine peri-operative monitoring. After the procedure each patient was individually observed, and the patient-specific chart of dynamic changes in pain perception was recorded, using the five-level Verbal Rating Scale (VRS). The multimodal analgesic treatment after THR was defined by the operating surgeons after considering indications and contraindications to the use of different groups of drugs (opioid and non-opioid analgesics). Postoperative pain was controlled by the patient-controlled analgesia method and VRS during the day-time, as well as night-time nurse-controlled analgesia. All medication adjustments were recorded in the individual patient files. In the case of moderate pain intensity (VRS-assessed), a patient was administered the non-opioid analgesic drug, and for high intensity pain the opioid. The analysis of pain relief therapy included information on the drugs applied, mode of dosing (single or multiple), daily dose, route of administration, and drug refusal due to the absence of pain recorded each study day, i.e. on the day of surgery and recovery in the postoperative room (day 0), and then daily from day 1 to day 6. Polymorphism rs1800795:G>C in the promoter region of the *IL6* gene (–174G>C) was determined using the PCR–RFLP method.

**Results:**

The patients carrying at least one *IL6* –174G allele (GG homozygote and GC heterozygote) were administered opioids significantly more often on days 0 (*p* = 0.0029), 3 (*p* = 0.019) and 4 (*p* = 0.031) after surgery compared with CC homozygous patients. Those patients also required a significantly higher opioid dose on days 3 (*p* = 0.029) and 4 (*p* = 0.030). Multivariate analysis demonstrated that the presence of the –174G allele was an independent factor predisposing patients to the administration of opioids during the first 24 h [*p* = 0.001, odds ratio (OR) 7.1, 95 % confidence interval (CI) 2.17–22.7], on day 3 (*p* = 0.01, OR 2.79, 95 % CI 1.25–6.26) and day 4 (*p* = 0.01, OR = 2.61, 95 % CI 1.17–5.79).

**Conclusion:**

The presence of the G allele *IL6* gene (–174G>C) polymorphism was found to be an independent factor predisposing to a higher dose and more frequent administration of opioids in the first days after total hip replacement.

## Introduction

Osteoarthritis (OA) is considered to be the main cause of persistent musculoskeletal pain and long-lasting invalidity. Hip joint OA is associated with chronic nociceptive pain, synthesis of proinflammatory cytokines [e.g. interleukin (IL)-1, IL-6, IL-8] and growth factors playing a major role in its pathophysiology [1, 2]. The first stage of pain treatment in OA includes non-steroidal anti-inflammatory drugs (NSAIDs) and acetaminophen (paracetamol). Alternatively, opioid analgesics or intra-articular glucocorticoids are also used [3]. Patients with advanced OA require surgical treatment. Regular analgesic treatment after total hip replacement (THR) includes the following drugs: opioids, acetaminophen, metamizole and NSAIDs. When these drugs are used in multimodal therapy, they produce better analgesic responses due to their synergistic actions [4]. Despite undeniable progress in pain relief pharmacotherapy, many patients do not receive appropriate and effective therapy. In recent years, increasing attention has been paid to the contribution of genetic factors to interpatient variability in the pain threshold and the efficacy of pain management. One of the postulated genes implicated in pain pathology and treatment response is interleukin 6 (*IL6*). The IL-6 protein is a multifunctional cytokine that plays an important role in a wide range of biological processes. The production and concentration of IL-6 can be influenced by the –174G>C functional genetic polymorphism in *IL6*. In vitro studies have demonstrated the influence of the polymorphism on IL-6 mRNA levels. –174C is associated with lower IL-6 levels and a reduced response to lipopolysaccharide stimulation. The in vitro findings were paralleled by in vivo observations; healthy –174C allele carriers are characterized by significantly lower levels of plasma IL-6 [5].

The aim of the present study was to evaluate the association between *IL6* (rs1800795: –174G>C) and opioid requirements in patients after THR.

## Methods

### Patients

The analysis of analgesic treatment was conducted in a group of 196 patients after THR (126 women, 61.2 %), aged from 32 to 86 years (mean 65.1 ± 10.7 years), during the planned 7 days of postoperative hospitalization. The mean body weight in the study group was 77.01 ± 14.56 kg, and the mean body mass index (BMI: kg/m^2^) was 27.1 ± 4.1. Of 196 study participants, 153 were non-smokers (78.1 %) and 43 (21.9 %) were smokers. The THR procedures were performed by three experienced orthopedic surgeons, via a standardized anterolateral approach using the same non-cemented implant system (Rimcup, Exception, Biomet), and medicated according to multimodal therapy in compliance with postsurgical analgesic treatment recommendations involving oral and/or parenteral analgesics with different mechanisms of action, i.e. opioid analgesics (OP) (morphine, pethidine and tramadol), non-opioid analgesics (acetaminophen and metamizole) and NSAIDs (ketoprofen and diclofenac) [6].

All patients were assessed by a consultant anaesthetist 24 h prior to the operation. To eliminate the impact of pharmacological treatment before the surgery on the results of the present study, all patients received the same type of premedication, 7.5 mg midazolam. Other analgesic administrations in 48 h before THR constituted one of the exclusion criteria. The THR procedure was performed using spinal anaesthesia after implementing routine peri-operative monitoring. Spinal anaesthesia was performed by an experienced consultant anaesthetist, after applying local anaesthetic to skin (2–5 ml of 1 % lidocaine), using the midline approach, with a pencil-point 27G spinal needle. After administration of 2–4 ml of 0.5 % heavy bupivacaine (Marcaine Spinal 0.5 % Heavy) without the use of any adjuvant, the level of the block was established prior to skin incision. The conduct of the spinal anaesthesia was recorded in the anaesthetic chart and was performed at the discretion of the anaesthetist. After the procedure patients were transferred to the postoperative ward and monitored according to local protocol. Each patient was individually observed, and the patient-specific chart of dynamic changes in pain perception was recorded, using the five-level Verbal Rating Scale (VRS). All patients, in the timeframe of 3–6 h after the surgery, were intravenously administered one of two (based on drug-related indications and contraindications) non-opioid analgesics, viz. 1.0 g metamizole or 1.0 g acetaminophen, as recommended by the Polish Association for the Study of Pain [7]. Patients were transferred to the orthopaedic ward immediately after successful pain control, i.e. no pain or mild pain assessed by VRS. The multimodal analgesic treatment after THR was defined by the operating surgeons after considering indications and contraindications to the use of the two groups of drugs (opioid and non-opioid analgesics). Postoperative pain was controlled by the patient-controlled analgesia (PCA) method and VRS during day-time, as well as night-time nurse-controlled analgesia (NCA). The pain intensity was recorded several times a day by a managing physician (at least during morning and evening check visits) or night-shift physician, complemented by recorded patients’ claims of increased pain intensity. In our study, rehabilitation started on the day of surgery, after discharge from the recovery room. To assess pain intensity, the five-level (VRS) was used, where a score of 3–4 was classified as a moderate intensity of pain, and score 5 as severe pain. The selection of opioids and non-opioids was based on the VRS score. In the case of moderate pain intensity (VRS 3–4), a patient was administered a non-opioid analgesic drug, and for high-intensity pain an opioid. All medication adjustments were recorded in the individual patient files. The dose of injectable opioids was converted to injectable morphine dose according to the conversion factors given in the literature: tramadol 10:1 and pethidine 10:1 [8]. For analysis of non-opioid and/or NSAID use, the 0/1 system was applied. The term 1 indicates a day on which non-opioids and/or NSAIDs were taken, whereas a day without medication with those drugs was described as 0. The simplified system was applied, since different drugs were administered (ketoprofen, diclofenac, acetaminophen, metamizole) and no reliable data is available to date for dose equivalence calculations. The above-mentioned medications were administered parenterally (opioids) or orally (non-opioid analgesics and NSAIDs). The analysis of pain relief therapy included information on the drugs applied, mode of dosing (single or multiple), daily dose, route of administration, and drug refusal due to the absence of pain recorded each study day. i.e. on the day of surgery and recovery in postoperative room (day 0), and then daily from day 1 to day 6. The protocol of the study was approved by the Ethics Committee at Pomeranian Medical University (BN-001/6/07), and all patients gave written informed consent.

### Genetic analysis

Genomic DNA was extracted from 200 μL of whole blood samples using Gene-MATRIX Quick Blood DNA Purification Kit (EURx, Poland). Polymorphism rs1800795:G>C in the promoter region of the *IL6* gene (–174G>C) was determined using the PCR–RFLP method, as described previously [9], with minor modifications. Briefly, a 299-bp fragment of the *IL6* gene was amplified during 35 cycles of PCR (initial denaturation at 95° for 5 min, then 95 °C/59 °C/72 °C, each step for 30 s) with the primer pair: sense primer: 5′-TGT CAA GAC ATG CCA AGT GCT-3′ and antisense: 5′-GCC TCA GAG ACA TCT CCA GT CC-3′. Amplification reactions were performed in a total volume of 12 μL using Mastercycler ep Gradient S (Eppendorf, Germany). The amplification mix contained 0.3 pmol/μL of each primer, 20 ng of genomic DNA, 0.6 U of RedTaq Polymerase (Sigma-Aldrich, Germany) in 1× enzyme-specific buffer (containing magnesium chloride at a final concentration of 1.5 mMol) and deoxynucleoside triphosphates (Sigma-Aldrich), 200 μmol of each. Subsequently, the PCR product was digested with *Hin1*II endonuclease (ThermoFisher Scientific, Lithuania) at 37 °C and separated on a 4 % agarose gel to distinguish between allele G (227 + 50 + 13 bp) and allele C (118 + 109 + 50 + 13 bp).

### Statistical analysis

The Mann–Whitney test was used to compare opioid doses between genotype groups. The *χ*^2^ test and Fisher exact test were used to analyze associations between genotypes or alleles and administration of opioids. Multiple logistic regression was performed to find independent variables associated with the administration of opioids. *p* < 0.05 was considered significant. Statistica 10 was used for statistical calculations.

## Results

The analysis of analgesic treatment revealed that 179 patients (91.33 %) required administration of opioids as a multimodal post-surgical analgesia on the day of the surgery. Opioids were not administered in 17 patients (8.67 %) due to a lack of pain, based on the verbal rating scale (VRS). The number of patients who continued opioid-based treatment in the subsequent days of hospitalization gradually decreased to 143 (72.95 %), 125 (63.77 %), 102 (52.04 %) and 59 (30.10 %) patients in the third, fourth, fifth and sixth day of treatment, respectively. Table 1 provides the mean doses (per kg of body weight) of opioids used on days 0–6, revealing a gradual reduction in demand for daily doses of opioids in the days following surgery. During the 7 days of hospitalization, 172 (87.75 %) received NSAIDs and 144 (73.46 %) were administered paracetamol.

**Table 1:** Opioid dose in the days following surgery

Day	Mean ± SD (mg/kg bw)
0	0.17 ± 0.10
1	0.21 ± 0.14
2	0.16 ± 0.12
3	0.14 ± 0.12
4	0.13 ± 0.13
5	0.08 ± 0.11
6	0.05 ± 0.09

*bw* body weight

Thirty-four patients were carriers of the *IL6*CC genotype (17.35 %), 93 patients (47.45 %) were heterozygous, and 69 (35.20 %) patients were carriers of the GG genotype. The distribution of *IL6* genotypes was in agreement with Hardy–Weinberg equilibrium (HWE *p* = 0.77). *IL6*CC homozygotes required a significantly lower opioid dose (per kg body weight) compared to GC + GG on day 3 (*p* = 0.029) and 4 (*p* = 0.030) (Table 2).

**Table 2:** Association of the *IL6* –174 gene polymorphism with opioid dose

Day	*IL6* –174G>C genotype			CC vs GC + GG
	GG (*n* = 69)	GC (*n* = 93)	CC (*n* = 34)	
	Mean ± SD (mg/kg bw)			p*
0	0.17 ± 0.10	0.18 ± 0.09	0.15 ± 0.13	0.160
1	0.19 ± 0.13	0.23 ± 0.14	0.20 ± 0.13	0.600
2	0.16 ± 0.13	0.17 ± 0.12	0.15 ± 0.12	0.600
3	0.14 ± 0.12	0.15 ± 0.11	0.10 ± 0.11	**0.029**
4	0.13 ± 0.13	0.13 ± 0.13	0.09 ± 0.12	**0.030**
5	0.07 ± 0.11	0.10 ± 0.11	0.06 ± 0.08	0.410
6	0.04 ± 0.09	0.06 ± 0.10	0.03 ± 0.06	0.320

Bold type indicates significantly different at *p* < 0.05

*bw* Body weight

* Mann–Whitney test

Table 3 presents the results of investigation on the potential association between *IL6* genotype and absence of opioid administration. The patients carrying at least one G allele (GG homozygote and GC heterozygote) were administered opioids significantly more often on days 0, 3 and 4 compared with CC homozygous patients. The frequency of the G allele was higher among patients who required opioid administration, but the difference did not reach statistical significance (Table 3).

**Table 3:** Comparison of *IL6* genotype and allele distribution in patients stratified by the need for opioid administration

Day	*IL6* –174G>C	Patients not administered opioids		Patients administered opioids		*p* value^a^		*p* value^b^	OR (95 % CI)
		*n*	%	*n*	%				
**0**	Genotype								
	CC	8	47.06	26	14.52	**0.0015**	GG + GC vs CC	**0.0029**	5.23 (1.85–14.79)
	GC	3	17.65	90	50.28		GG vs GC + CC	1.000	1.00 (0.35–2.82)
	GG	6	35.29	63	35.20		GG vs CC	0.063	3.23 (1.02–10.23)
	Allele								
	G	15	44.12	216	60.34				
	C	19	55.88	142	39.66		G vs C	0.071	1.93 (0.95–3.92)
**3**	Genotype								
	CC	15	28.30	19	13.29	**0.039**	GG + GC vs CC	**0.019**	2.58 (1.19–5.55)
	GC	20	37.74	73	51.05		GG vs GC + CC	0.870	1.08 (0.56–2.09)
	GG	18	33.96	51	35.66		GG vs CC	0.076	2.24 (0.94–5.31)
	Allele								
	G	56	52.83	175	61.19				
	C	50	47.17	111	38.81		G vs C	0.170	1.41 (0.90–2.21)
**4**	Genotype								
	CC	18	25.35	16	12.80	0.074	GG + GC vs CC	**0.031**	2.31 (1.09–4.89)
	GC	29	40.85	64	51.20		GG vs GC + CC	0.880	1.10 (0.60–2.03)
	GG	24	33.80	45	36.00		GG vs CC	0.091	2.11 (0.91–4.87)
	Allele								
	G	77	54.23	154	61.60				
	C	65	45.77	96	38.40		G vs C	0.170	1.35 (0.89–2.05)

Bold type indicates significantly different at *p* < 0.05

*OR* odds ratio; *CI* confidence interval

^a^ *χ* ^2^ test; ^b^ Fisher exact test

Multivariate logistic regression analysis was performed, with age, sex, BMI, tobacco smoking status, NSAIDs, acetaminophen, and the –174G allele as the independent variables. The analysis demonstrated that the presence of the –174G allele was an independent factor predisposing patients to administration of opioids during the first 24 h [*p* = 0.001, odds ratio (OR) 7.1, 95 % confidence interval (CI) 2.17–22.7], on day 3 (*p* = 0.01, OR 2.79, 95 % CI 1.25–6.26) and day 4 (*p* = 0.01, OR 2.61, 95 % CI 1.17–5.79) after THR. In the same multivariate analysis, administration of NSAIDs significantly influenced the need for opioid analgesics only in the first 24 h of the postoperative period (the day 0, *p* = 0.02).

## Discussion

Total hip replacement involves trauma to soft and bony tissues, and can result in considerable pain. It is worth noting that the greater the amount of tissue injury, the more active the inflammation that can occur in the perisurgical region. After tissue injury, a proinflammatory cytokine, IL-6, is overproduced by many cells throughout the body [10, 11]. Higher IL-6 concentrations trigger synthesis of acute phase proteins such as CRP, and activation of the innate immune system [12]. Since the *IL6* –174G allele is associated with increased gene expression, it may lead to even higher IL-6 levels in the allele carriers, resulting in increased inflammation and, as a consequence, greater stimulation of nociceptors by mediators generated in the inflammatory zone. The results of the current study are consistent with that hypothesis, as the presence of the G allele predisposed patients to higher demands for analgesics, i.e. opioid requirements in the early postoperative period.

This observation is contrary to the results of Reyes-Gibby et al. [13], who reported that lung cancer patients with the *IL6* –174CC genotype required significantly higher daily opioid doses than carriers of the G allele. However, the aforementioned study involved patients on previous opioid medication and with chronic pain, contrary to our study. Unfortunately, the current study did not involve measurements of IL-6 concentrations. However, it was previously demonstrated that the IL-6 level increases after THR, and the peak concentrations after operation occur at 12–24 h [11]. Considering the above-mentioned observations, it may be assumed that IL-6 concentrations were highest on the first day after the surgery. The *IL6* –174GG genotype patients could be characterized by higher IL-6 levels compared with CC carriers, with resultant increased demands for opioids within 24 h of the surgery. The observation of the present study indicated that morphine doses and pain intensity gradually decreased during the postoperative period, but a subgroup of patients still suffered from pain of significant intensity. The patients carrying at least one *IL6* –174G allele (GG homozygote and GC heterozygote) were administered opioids significantly more often, also on days 3 and 4, compared with CC homozygous subjects. One possible explanation may be derived from in vitro studies, which demonstrated peripheral actions of opioids such as modulation of inflammatory processes and wound healing [14]. Opioids can interfere with different stages in the inflammatory cascade evoked by tissue injury, trauma or infection, mitigating the inflammation. However, adding those findings to the results of the present study, we may assume that patients predisposed by genetic factors (determined by *IL6* –174G>C polymorphism) are characterized by elevated levels of IL-6, and thus require a more intensive analgesic approach in the early postoperative period. It is known that IL-6 levels decrease gradually after surgical procedures [11, 15], as IL-6 production is primarily regulated by a negative feedback mechanism through suppressors of cytokine signaling molecules, coded by genes of the Janus kinase and signal transducer and activator of transcription (JAK-STAT) pathway. This, in turn, reduces overproduction of IL-6 after injury.

Our study also demonstrated that administration of NSAIDs significantly influenced the need for opioid analgesics within the first 24 h of the postoperative period. This observation is in keeping with the report of Slattery et al. [16], who demonstrated correlation between the analgesic effects of acetylsalicylic acid and *IL6* genotype in breast cancer patients. Acetylsalicylic acid effects were most potent in female carriers of the wild-type *IL6* –174G allele, which was associated with constitutively high IL-6 levels. It is worth noting that in the multivariate logistic regression analysis the presence of the –174G allele was an independent factor predisposing patients to administration of opioids during the first 24  and, on days 3 and 4 after THR.

The results of the present study may be biased by some confounding factors, especially by factors other than genetic ones which can affect the circulating IL-6 concentrations (e.g. other cytokines that have been shown to modulate pain), and gene polymorphisms of other pain modulators, not assessed in this study. Because of the complexity of pain pathology and inflammatory responses, most likely combinations of multiple polymorphisms would be adequate as predictors of analgesia.

## Conclusion

The *IL6* (rs1800795: G>C) –174 gene polymorphism has a significant influence on the overall requirement and daily doses of opioids in patients after elective THR.
